# Effectiveness of Shugan Jieyu capsules for psychiatric symptoms of epilepsy: a systematic review and meta-analysis

**DOI:** 10.1186/s12906-024-04361-0

**Published:** 2024-01-29

**Authors:** Sejin Kim, Yunna Kim, Seung-Hun Cho

**Affiliations:** 1 College of Korean Medicine, Kyung Hee University, 23, Kyungheedae-ro, Dongdaemun-gu, Seoul, 02447 Republic of Korea; 2 Department of Neuropsychiatry, College of Korean Medicine, Kyung Hee University Medical Center, Kyung Hee University, Seoul, 02447 Republic of Korea; 3 Research group of Neuroscience, East-West Medical Research Institute, WHO Collaborating Center, Kyung Hee University, Seoul, 02447 Republic of Korea

**Keywords:** Epilepsy, Shugan Jieyu capsule, Psychiatric symptoms, Depression, Anxiety, Insomnia, Systematic review, Meta-analysis

## Abstract

**Background:**

The relationship between epilepsy and depression is bidirectional. One condition exacerbates the other. However, there are no current guidelines for treating depression in epilepsy patients. In some cases, seizures worsen when antidepressants (AD) are prescribed or when they are discontinued due to adverse events. The Shugan Jieyu capsule, composed of *Acanthopanax senticosus* and *Hypericum perforatum*, is a widely used herbal medicine for treating depression. This study aimed to explore the effectiveness and safety of Shugan Jieyu capsules (SJC) in relieving depression in patients with epilepsy.

**Methods:**

We searched English, Korean, Japanese, and Chinese databases in October 2023 to collect all relevant randomized clinical trials (RCTs). The primary outcomes were the depression scale scores and seizure frequency. The secondary outcomes were quality of life (QoL) and adverse events.

**Results:**

Nine RCTs were included in this meta-analysis. Compared with AD, SJC showed significant differences in the improvement of depression (SMD: 3.82, 95% CI: 3.25, 4.39) and reduction in seizure frequency (MD: 0.39 times/month, 95% CI: 0.28, 0.50). SJC showed more beneficial results than antiepileptic drugs (AED) in terms of antidepressant effects (SMD: 1.10, 95% CI: 0.69, 1.51) and QoL (MD: 11.75, 95% CI: 10.55, 12.95). When patients were prescribed AED, the additional administration of SJC improved depression symptoms (SMD: 0.96, 95% CI: 0.28, 1.63). The SJC treatment group had a lower incidence of side effects than the control group. However, the difference was not statistically significant.

**Conclusions:**

Our results suggest that SJC may be effective in treating depression in patients with epilepsy. Additionally, SJC has the potential to help reduce seizure frequency in epilepsy patients with depression.

**Supplementary Information:**

The online version contains supplementary material available at 10.1186/s12906-024-04361-0.

## Background

Epilepsy is a chronic pathological condition of the brain that causes recurrent seizures. Epilepsy is diagnosed when a minimum of two unexplained seizures occur at least 24 h apart [1]. Approximately 2–5% of the general population suffer from seizures [2]. One-third of these patients eventually develop epilepsy. The overall lifetime prevalence is 7.60 per 1,000 people [3]. Although most epilepsy cases are treatable and enter a period of prolonged seizure remission, one-third to one-half of cases develop treatment-resistant epilepsy [4]. Patients with refractory epilepsy are more likely to experience prolonged recurrent seizures and a state of epileptic overlap. This increases their risk of injury and sudden death [5]. In addition, refractory epilepsy lowers their QoL and causes serious neuropsychological, psychological, and social impairments. Unfortunately, there is no current treatment that can resolve these conditions.

A recent systematic review and meta-analysis reported that patients with epilepsy have a higher risk of developing depression. Depressed patients have a higher risk of developing three to seven various types of epilepsy [6, 7]. The relationship between epilepsy and psychiatric symptoms is bidirectional. Both epilepsy and depression share mechanisms associated with hyperactivity of the hypothalamic-pituitary-adrenal axis. They also share impairment of neurotransmitter systems, mainly the neurotransmitters serotonin and norepinephrine [8]. Clinically, epilepsy can influence the onset of depression through exposure to chronic stress [9]. In addition, as seizures recur, they become fixed in the form of learned helplessness. This eventually leads to depression [10]. Conversely, depression can increase seizure frequency directly through the mechanism of sleep deprivation and often interferes with the activity of antiepileptic drugs (AED) [11]. Depression is a serious comorbidity of epilepsy, which induces a vicious cycle. Therefore, treatment of both depression and epilepsy is essential.

However, there are no high-quality guidelines for specific antidepressants (AD) or AED that may be useful specifically for patients with epilepsy and depression [12]. The use of antidepressants in epilepsy remains controversial, although the treatment of depression with selective serotonin reuptake inhibitors (SSRIs) is often prescribed. In general, it is believed that when taken together with AED, low or therapeutic SSRI doses could be sufficient to induce seizures [13]. However, according to a recent meta-analysis of patients with partial epilepsy and depression, SSRI treatment did not significantly increase seizure frequency [14]. It is also noteworthy that patients treated with antidepressants tend to withdraw from antidepressants due to their side effects rather than their ineffectiveness. Reported side effects of SSRIs include nausea, dizziness, sedation, gastrointestinal disturbances, and sexual dysfunction [15].

Therefore, in situations where effective and safe drugs with reliable evidence are required, herbal medicines must also be considered. The Shugan Jieyu capsule (SJC), composed of *Acanthopanax senticosus* and *Hypericum perforatum* [16], has been licensed since 2008 and is commonly used to treat depression. According to clinical trials, the antidepressant effect of SJC was like that of escitalopram, an SSRI. SJC induced fewer side effects than escitalopram [17]. *Acanthopanax senticosus* regulates the central nervous and immune systems and protects neurons [18]. The water-based extract of *Acanthopanax senticosus* significantly reduced the immobility of mice in the forced swimming test and tail suspension test in vivo [19]. *Hypericum perforatum*, also known as St. John’s wort, is clinically effective against depression [20]. It was superior to a placebo in patients with major depression and was as effective as a tricyclic or tetracyclic antidepressant or SSRI [21]. Moreover, adverse events occurred less frequently in patients treated with *Hypericum* than in patients receiving standard AD [22].

There has been one report on the effect of SJC on epilepsy. *Acanthopanax senticosus* has been reported to have sedative effects in preclinical studies on rodents [23]. In a mouse model, ethanol and water-based extracts of *Hypericum perforatum* increased the latency of pentylenetetrazole-induced convulsions in a dose-dependent manner [24]. However, in the rabbit model, it was confirmed that the polar fraction (water, n-butanol) inhibited epileptic seizures. On the other hand, the non-polar ether fraction enhanced epilepsy [25].

In recent years, along with experimental studies on its’ efficacy in each disease, there have been numerous RCTs on SJC in patients with epilepsy and depression. In addition, certain conflicting results regarding the impact of SJC on seizures have been reported. Therefore, high-quality evidence to evaluate the clinical efficacy and safety is lacking. Here, we provide evidence for the clinical use of SJC in epilepsy with depression through a systematic review and meta-analysis.

## Methods

The protocol for this systematic review was registered in the International Prospective Register of Systematic Reviews (PROSPERO). The trial registration number is CRD 42,021,238,804. No ethical approval was necessary as this was a systematic review. The systematic review and meta-analysis reporting the effectiveness of Shugan Jieyu capsules for psychiatric symptoms of epilepsy was reported according to the Preferred Reporting Items for Systematic Reviews and Meta-Analyses (PRISMA) guidelines, and the PRISMA checklist was attached as Additional File 1.

### Search strategy and selection

Two reviewers (YNK and SJK) conducted a thorough literature investigation of the following online databases from inception to October 2023: PubMed, EMBASE, Cochrane Central Register of Controlled Trials, Cumulative Index to Nursing and Allied Health Literature(CINAHL), Korean Medical Database, OASIS, Korean Traditional Knowledge Portal, CiNii, and China National Knowledge Infrastructure(CNKI). Search terms were adapted to suit each database. For PubMed, we used the search strategy as follows: (“epilepsy“[Mesh] OR “epilep*”[Title/Abstract] OR “seizures“[Mesh] OR “convulsion”[Title/Abstract] OR “seizure*“[Title/Abstract]”) AND (“Shugan jieyu” [Title/Abstract] OR “Shugan jieyu capsule”[Title/Abstract] OR “Shuganjieyu”[Title/Abstract] OR “Shugan-jieyu”[Title/Abstract] OR [(“Acanthopanax senticosus”[tw] OR “Eleutherococcus senticosus”[tw] OR “Siberian Ginseng”[tw]) AND (“*Hypericum perforatum*”[tw] OR “John’s wort”[tw])] OR “舒肝解郁”[tw] OR “舒肝解郁胶囊”[tw] OR (“贯叶金丝桃”[tw] AND (“刺五加”[tw] OR “五加皮”[tw]). Detailed search strategy for each database was provided in Additional file 2.

After the initial search, we included all studies identified in the databases in EndNote X9 software for data selection. Both reviewers had the ability to read papers in English, Korean, Chinese, and Japanese. Candidate studies were selected according to the following criteria.

The selected studies included:

(1) Randomized control trials (RCTs) of the SJC for the treatment of psychiatric symptoms in patients with epilepsy; (2) patients diagnosed with epilepsy (any type), patients who met any criteria for each psychiatric symptom (e.g., depression, anxiety, insomnia) in a questionnaire or validated tool, such as the Patient Health Questionnaire 9-item Depression and Hamilton Depression Rating Scale. There were no restrictions with respect to sex, age, or ethnicity. (3) investigation of the efficacy of the SJC and modified SJC. The SJC is composed of *A. senticosus* and *H. perforatum* [16]. The modified SJC was also included. It is prescribed according to personal differentiation by adding to or subtracting from the original herbs. The modified SJC has approximately the same effect as the original prescription.

Studies were excluded if they met any of the following criteria: (1) did not use outcome measures of interest; (2) reported duplicate data.

**Fig. 1:**
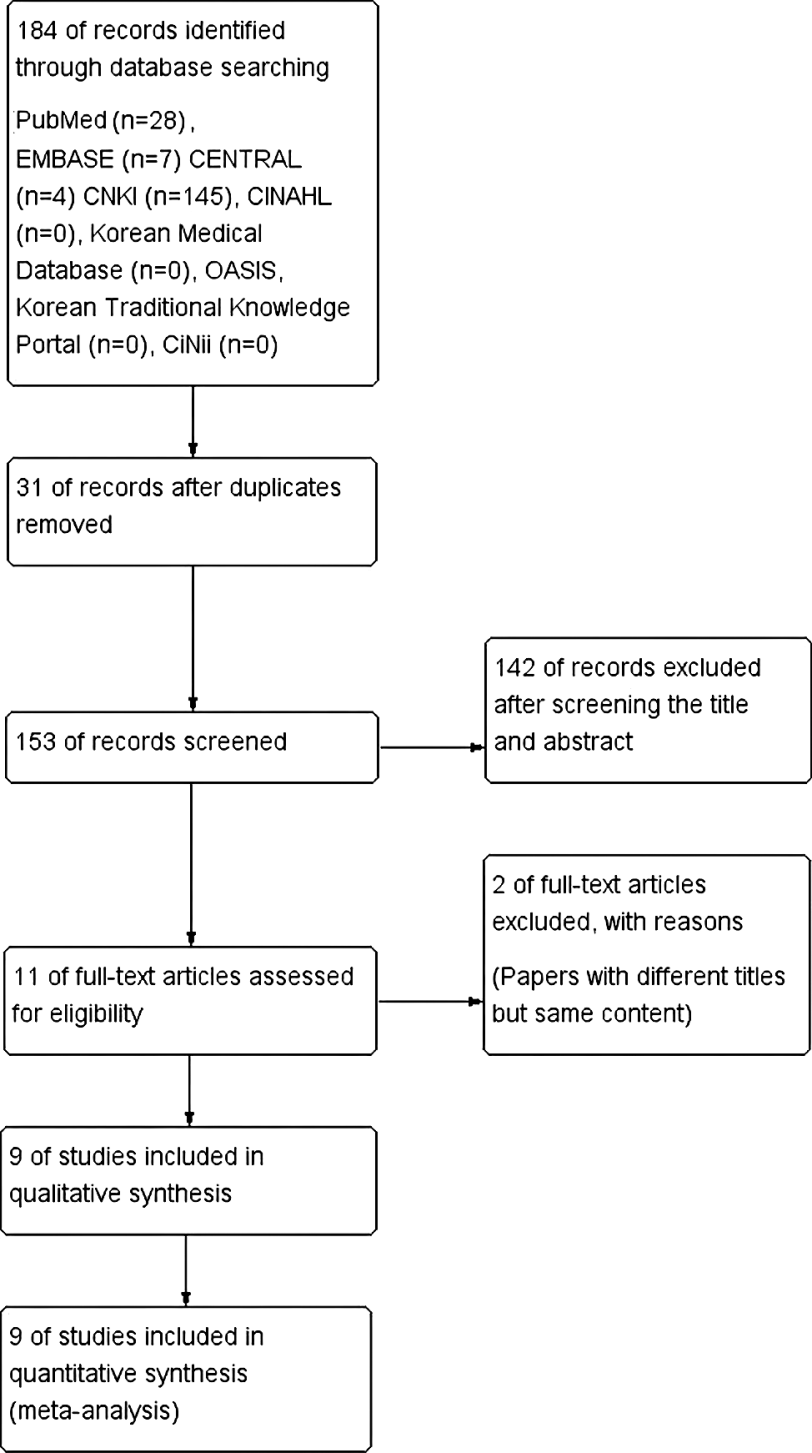
Flow diagram of the study

Two reviewers completed the selection process. We reviewed the titles, abstracts, and studies to ensure that they met the criteria stated above. The full text was obtained for all potentially eligible studies, and eligibility was assessed independently. Any disagreement regarding the eligibility of a study was resolved through discussion. The flow diagram of the selection process is shown in Fig. 1.

### Data extraction and items

Data extraction was performed according to an established protocol. The extracted data included details of the author, publication year, participant characteristics, setting, intervention, treatment period, controls, outcome measures, and adverse events. One reviewer (SJK) conducted a full abstraction of all the data. The accuracy of the data was then verified by two reviewers (SJK and YNK).

### Assessment of risk of bias

Two reviewers (SJK and YNK) independently assessed the risk of bias using the Cochrane Risk of Bias Assessment Tool (RoB 2.0) [26]. The methodological quality of the included studies was evaluated based on the following five aspects: bias arising from the randomization process, bias due to deviations from intended interventions, bias due to missing outcome data, bias in the measurement of the outcome, and bias in the selection of the reported result. Finally, we decided on the overall risk of bias by combining these five aspects.

Each aspect was categorized as a low risk of bias, high risk of bias, or as having “some concerns.” If all factors that make up each aspect were at low risk of bias, the aspect was judged as “low risk of bias.” If there is some concern in at least one factor, but there was no high risk of bias, the study was judged to have “some concerns.” If there was a high risk of bias in at least one factor or there were concerns in multiple factors, the study was judged to be “high risk of bias.” We judged that if some concern emerged in three or more areas, it lowers confidence in the result, so we set the overall risk of bias as high.

### Outcome measures and statistical analysis

The primary outcome measures were scores on scales for depression, such as the Hamilton Depression Rating Scale, Chinese-Neurological Disorders Depression Inventory for Epilepsy, Center for Epidemiological Studies-Depression Scale and Patient Health Questionnaire 9-item Depression. In addition, we analyzed the frequency of monthly epileptic seizures to identify antidepressants with the lowest risk of seizure exacerbation. The quality of life (QoL) was also evaluated using the Quality of Life in Epilepsy Inventory 31 (QOLIE-31).

We used the difference between values before and after treatment to reduce the heterogeneity caused by differences in baseline and evaluation tools in continuous data. If only the values before and after treatment ​​were reported in the study, the mean and standard deviation of the amount of change were directly calculated.

$$\sqrt{{s.d.}_{pre}^{2}+{s.d.}_{post}^{2}-2\times {r}_{pre.post}\times {s.d.}_{pre}\times {s.d.}_{post}}$$

Continuous data were assessed using standardized mean differences (SMDs) or mean differences (MDs) with 95% confidence intervals (CIs), and dichotomous data were evaluated as an odds ratio (OR) with 95% confidence intervals (Cls). If there was high heterogeneity between studies, a standardized mean difference was adopted. In addition, despite the differences in study designs, we attempted to report the results based on a random-effects model, assuming that the relevant intervention effects were estimated.

All statistical analyses were conducted using the Cochrane Collaboration’s software program Review Manager (RevMAN) for Windows.

To assess statistical heterogeneity, an I^2^ test was conducted to determine the degree of heterogeneity regardless of the number of studies. Heterogeneity can be classified into four levels: no heterogeneity (0% ≤ I2 ≤ 25%), low heterogeneity (25% < I2 ≤ 50%), medium heterogeneity (50% < I2 ≤ 75%), and high heterogeneity (I2 > 75%) [27]. When I^2^ was < 50%, there was statistical homogeneity, where an overall estimate could be obtained by integrating the data from individual studies.

### Publication bias assessment

A funnel plot was used to evaluate publication bias. However, based on the small number of clinical studies, the ability of a funnel plot to detect bias may be limited [28].

### Certainty of evidence assessment

To assess the certainty of evidence for the effectiveness of Shugan Jieyu capsules included in the meta-analysis, we used Grading of Recommendations, Assessment, Development, and Evaluation (GRADE). The GRADE system is a methodology for determining the certainty of evidence using four grading levels: very low, low, medium, and high. The certainty of the evidence is determined by the research design, five factors that can lower the certainty of the evidence, and three factors that can increase it.

Because the nine articles included in our study were RCTs, the starting grade of certainty of evidence was high. The certainty of evidence was lowered according to the following five factors: (1) study limitation (high risk of bias present in the majority of the studies), (2) inconsistency (I2 > 70%), (3) indirectness (not clinically) assessed depression, frequency of seizure and QoL), (4) imprecision (range of the 95% CI > 2.0), and (5) presence of publication bias. The certainty of the evidence was lowered depending on the following three factors: (1) large effect size (the risk estimate of a risk factor > 2.5), and (2) presence of a dose-effect relationship in the reported study (3) presence of plausible residual confounding. Two authors independently assessed quality criteria and discussed them.

## Results

### Characteristics of included studies

A total of 184 studies were identified through nine electronic databases, four of which had more than one search result. We excluded 31 duplicates and screened 153 studies. A total of 142 irrelevant studies were excluded after screening titles and abstracts. Two records were excluded after the full text screening. Finally, 9 RCTs were assessed to be eligible after full text screening. The identification process of relevant studies is shown in Fig. 1.

The characteristics of the included studies are summarized in Additional File 3. All the studies were published between 2015 and 2023 and were conducted in China. Although the primary authors were different in the two studies, the contributing authors partially overlapped [29, 30].

### Participants

A total of 815 patients were included in the nine studies. All the participants were included in the statistical analysis. Seven studies included patients diagnosed with depression as well as epilepsy. Two study included patients diagnosed with depression and anxiety as well as epilepsy.

### Intervention and control

Two studies compared SJC with oxcarbazepine, an AED, and an AD which was unnamed, respectively [31, 32]. In two studies, while maintaining the AED which was originally taken, a group was administered SJC and was compared with the group that was not [30, 33]. SJC and AED were compared with AD and AED in three studies [29, 34, 35]. In one study, AED and sertraline that were previously administered were dispensed to the control group [29]. Sodium valproate and escitalopram were administered in another study [34]. In a different study, pre-existing AED and tandospirone were used in a control group [35]. Two study compared the combined administration of SJC, pre-existing AED, and AD, with the combined administration of AED and AD [36, 37]. The treatment duration ranged from two to 24 weeks.

### Outcome measures

In all enrolled studies, scales of depression were measured (e.g., HAMD, PHQ-9, CES-D, and C-NDDIE). Four studies used the HAMD [31, 32, 34, 36], three studies used the PHQ-9 [29, 30, 33], another study used CES-D [37], and the other study used the C-NDDIE [35]. One study simultaneously evaluated the HAMD and Zung Self-Rating Depression Scale (SDS) [34]. Five studies measured seizure frequency (per month), and five studies evaluated the QoL of patients with epilepsy. The QoL evaluation tool was consistent with the QOLIE-31.

### Methodological quality of the included studies

**Fig. 2:**
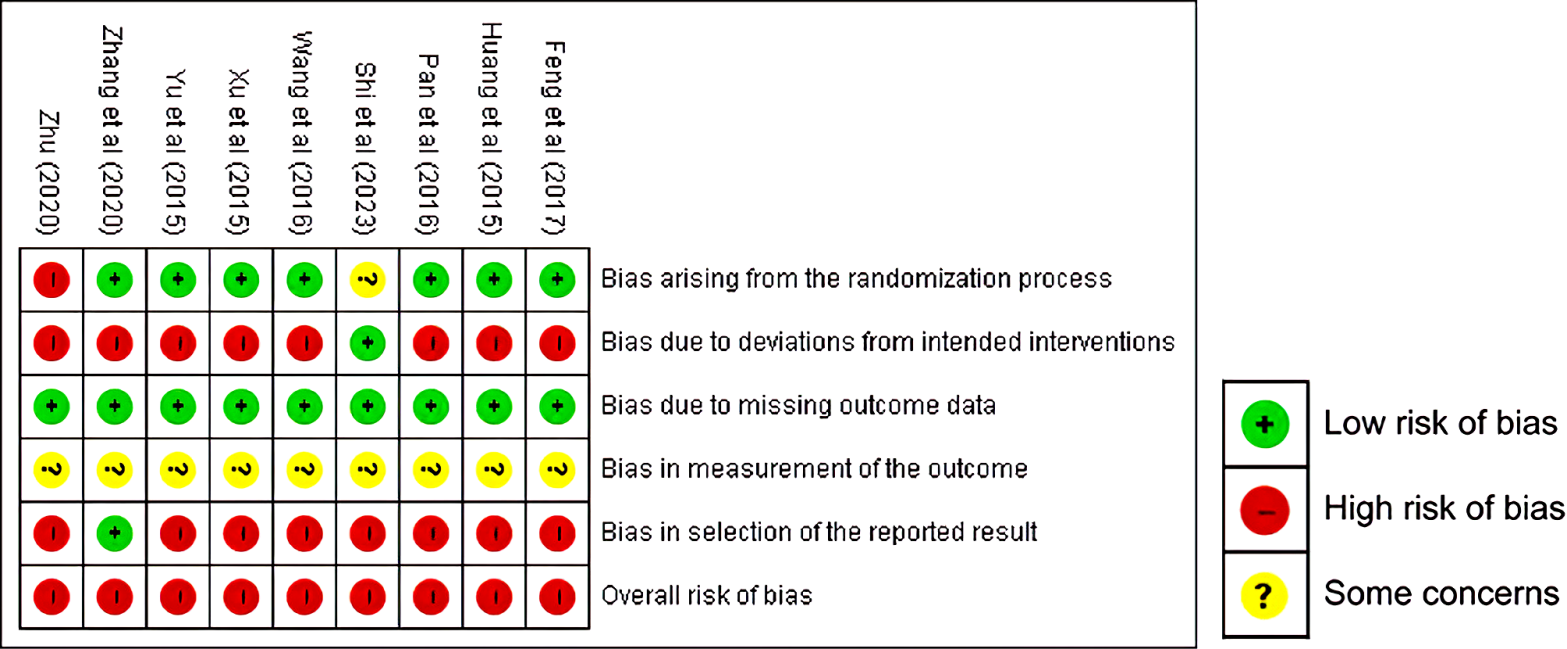
Risk of bias summary

The risk of bias assessed by the Cochrane Collaboration’s RoB 2.0 tool is shown in Fig. 2. All studies were randomized, except for one that was assigned according to the order of admission. In all studies, nearly all the intervention results were available. However, All but one study [37] were not performed blinding of the researchers, subjects, intervention providers, or caregivers. In addition, in all studies, there was no available information on the examiners of the reported outcomes. Due to insufficient information, bias in the measurement of the outcomes was evaluated as a concern. Only one study had a pre-registered clinical trial protocol [35]. The remaining eight did not. The absence of a clinical trial protocol can cause a high risk of bias in the selection of the reported results. The overall risk of bias in the five domains was determined to be a “high risk of bias,” as at least one of the five domains had a high risk of bias in all the studies.

### Depression

**Fig. 3:**
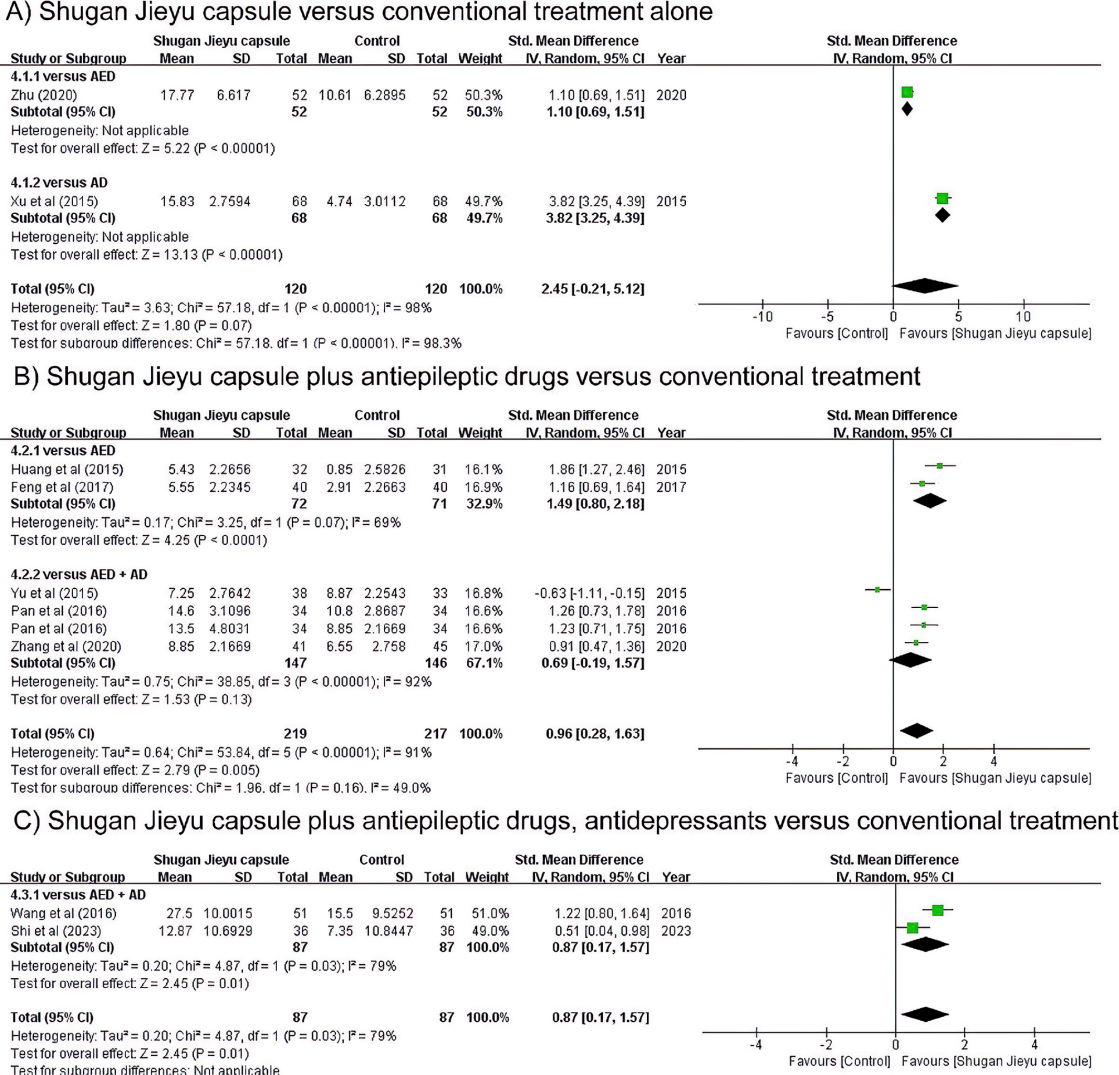
Forest plot illustrating the use of Shugan Jieyu capsules versus conventional treatment for depression

A positive effect of SJC alone on depression was reported in two RCTs (Fig. 3A). Each study measured HAMD changes at 18 and 12 weeks compared with prior treatment. SJC slightly reduced depression compared to AED alone [31]. In addition, SJC reduced depression compared with AD alone [32]. Taken together, there was no significant difference in the antidepressant effects between SJC administration and conventional treatment alone (SMD 2.45, 95% CI: -0.21, 5.12).

Five studies reported the effects of SJC plus AED in depression (Fig. 3B). Among them, two studies that compared the AED group used the PHQ-9 after 12 weeks [30, 33]. These studies reported the antidepressant effect of SJC, both individually and in combination (SMD 1.49, 95% CI: 0.80, 2.18). The three studies comparing AED plus AD treatment groups had different measurement scales: PHQ-9, HAMD, Zung SDS, and C-NDDIE. The PHQ-9 was measured before and after eight weeks of treatment [29]. The antidepressant activity of sertraline was higher than that of SJC when the conventional AED intake was maintained. In contrast, the antidepressant effect of SJC was higher than that of escitalopram after eight weeks when the existing AED dose was maintained [34]. In this study, HAMD and SDS were evaluated simultaneously. Therefore, we allocated half of the total number of subjects to both outcomes. In addition, SJC significantly reduced depression compared to tandospirone in the change before and after 12 weeks, as measured by the C-NDDIE [35]. However, according to the pooled results, there was no statistically significant difference in the effect of reducing depression between the AD and SJC groups when AED intake was maintained (SMD 0.96, 95% CI: -0.19, 1.57). Pooling the above studies, the administration of SJC while maintaining the existing AED administration had a beneficial antidepressant effect (SMD 0.96, 95% CI: 0.28, 1.63).

Two studies reported the effect of SJC plus AED plus AD [36, 37] (Fig. 3C). When evaluated by HAMD after 12 weeks of administration, depression levels were reduced compared to conventional AED plus AD treatment. Also, when 2 weeks of additional SJC medication was evaluated with CES-D, a significant effect was observed. Taken together, there was significant difference in the antidepressant effects when additional SJC was adminisered while taking SJC plus AED plus AD (SMD 0.87, 95% CI: 0.17, 1.57).

### Anxiety

There were two studies assessing anxiety in epilepsy patients, using the GAD-7 index [37, 38]. One study reported the anti-anxiety effect of SJC plus AED versus AD plus AED [38]. At 12 weeks, the score of GAD-7 in the treatment group was 3.67 ± 3.28 and the control group was 4.93 ± 3.58, showing a statistically significant difference between the two groups (*p* = 0. 094). The other study reported the anti-anxiety effect of SJC plus AED plus AD versus AED plus AD [37]. After two weeks of medication, the score of the treatment group was 4.17 ± 2.25 and the control group was 7.38 ± 3.31, and the statistical difference between the two groups was significant (*p* < 0.001).

When AED was maintained, administering SJC had a benefit of anti-anxiety effect than AD. Also, when AED and AD were maintained, additional SJC had a benefit in reducing anxiety.

### Frequency of seizures

**Fig. 4:**
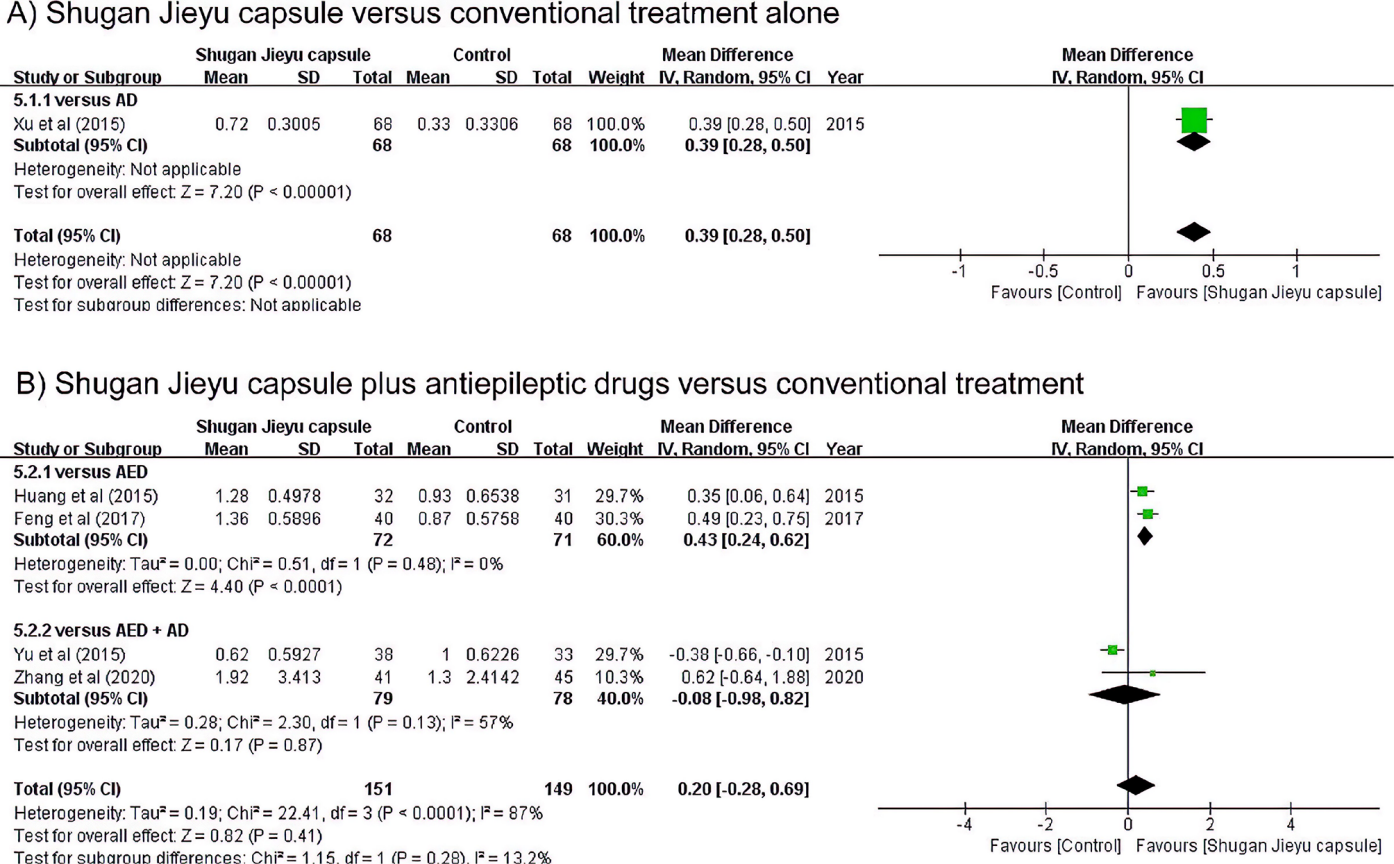
Forest plot illustrating the change pre- and post-treatment in the frequency of seizures between the use of Shugan Jieyu capsules and conventional treatment

The following forest plot shows the change from pre -to post-treatment. Larger values indicate a greater decrease in seizure frequency (Fig. 4).

One study compared the effects of SJC and AD on seizure frequency [32]. When administered for four weeks, SJC had a significantly lower seizure frequency than AD (Fig. 4A).

Four studies compared the effects of SJC on seizure frequency when AED was administered in combination (Fig. 4B). Two of these studies compared the seizure frequency before and after 12 weeks [30, 33]. Taken together, the seizure frequency significantly decreased when SJC was administered while AED was maintained (MD 0.43 times/month, 95% CI: 0.24, 0.62). Two additional studies compared the added administration of AD with SJC in the context of maintaining AED intake. Sertraline for 24 weeks [29] and tandospirone for 12 weeks were used as the controls [35]. When the two studies were combined, there was no significant difference in seizure frequency between SJC and AD administration when AED intake was maintained (MD -0.08 times/month, 95% CI: -0.98, 0.82). When a total of four studies were taken together, in the presence of pre-existing AED, SJC did not significantly affect seizure frequency (MD 0.20 times/month, 95% CI: -0.28, 0.69).

### Quality of life

**Fig. 5:**
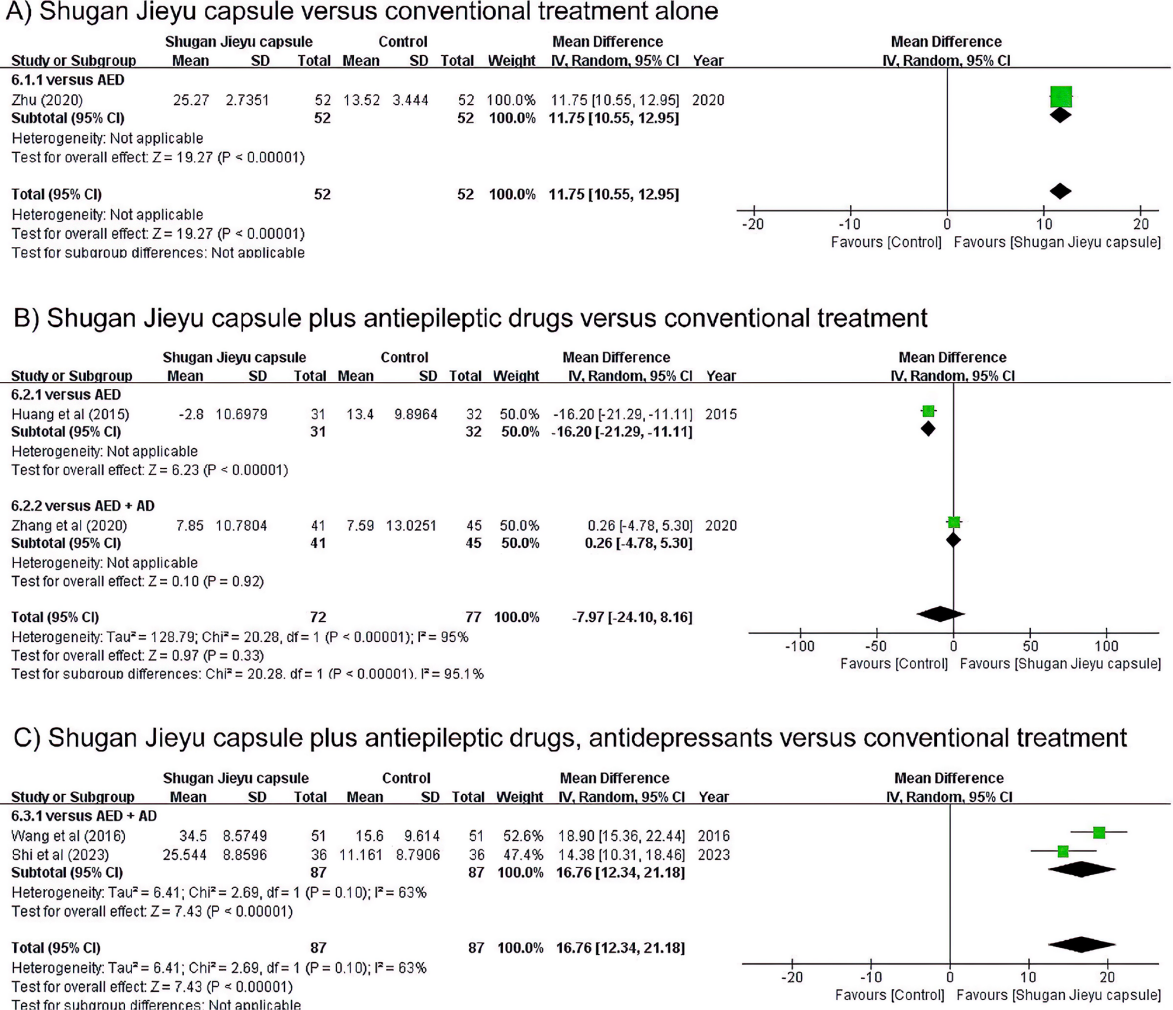
Forest plot illustrating Shugan Jieyu capsules versus conventional treatment in terms of quality of life

All studies that measured QoL used the QOLIE-31, a quality of life indicator specific to patients with epilepsy (Fig. 5). The group administered SJC for 18 weeks had a higher QoL than the group administered oxcarbazepine [31]. Two studies reported the effect of SJC on QoL when AED intake was maintained. Only one studies reported QoL changes when additional SJC was administered for 12 weeks, while the AED dose was maintained [30] (Fig. 5A). The increase in QoL was greater in the group that did not receive additional SJC. The difference in QoL between additional doses of SJC and AD was not significant [35]. Taken together, the benefit of taking SJC while maintaining AED intake was not statistically significant in the case of QoL (MD -7.97, 95% CI: -24.10, 8.16; Fig. 5B). There was a benefit in QoL when SJC was additionally administered, while AED and AD were administered for 2 weeks and 24 weeks [36, 37] (MD 16.76, 95% CI: 12.34, 21.18; Fig. 5C).

### Adverse events

**Fig. 6:**
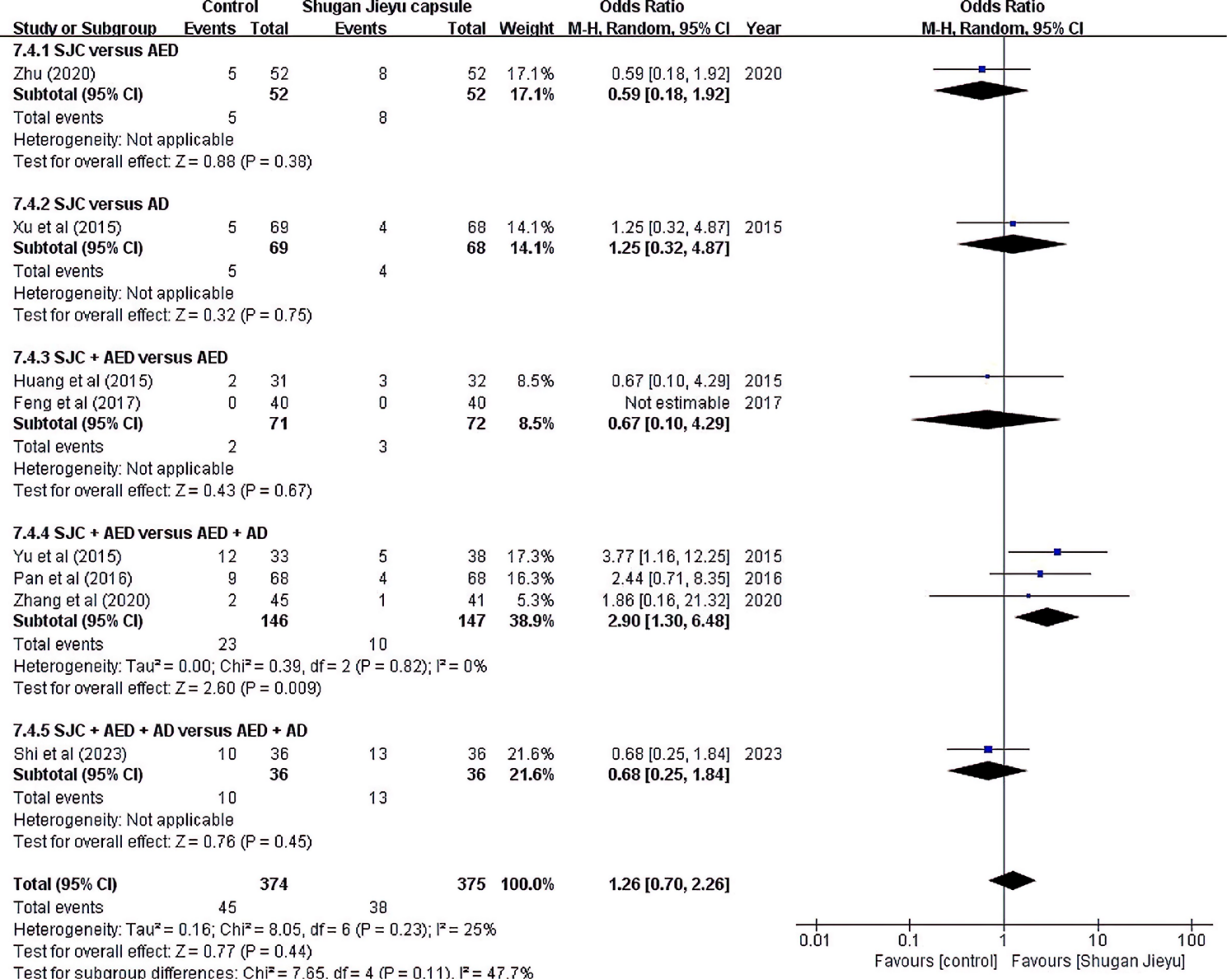
Adverse events

All adverse events reported in the eight studies are listed in Table 1. Of the 815 patients included in the RCTs, 104 adverse events were confirmed. A meta-analysis comparing the odds ratios for adverse events in the control and SJC treatment groups is shown in Fig. 6. Wang et al. did not report adverse events in the study and control groups. Therefore, these adverse events were not included in the meta-analysis.

**Table 1:** Adverse events

Ref	Sample size	Number of Adverse events	Rate of Adverse events (%)	Cardiovascular events	GI events	Neurological events	Physiological events
Huang et al. (2015)	E: 32 C: 31	E: 3 C: 2	E:9.38 C: 6.45		E: diarrhea (1), nausea (1)	E: dizziness (1) C: dizziness (1)	C: drowsiness (1)
Yu et al. (2015)	E: 38 C: 33	E: 5 C: 12	E: 13.2 C: 34.3		E: diarrhea & nausea (5) C: diarrhea (1), constipation (1)	C: dizziness (3), severe dizziness and vomiting (2)	C: drowsiness (3), dry mouth (2)
Xu et al. (2015)	E: 68 C: 69	E: 4 C: 5	E: 5.88 C: 7.35		E: diarrhea (2), nausea (1) C: diarrhea (1), nausea (2)	E: dizziness (1) C: dizziness (2)	
Pan et al. (2016)	E: 68 C: 68	E: 4 C: 9	E: 5.9 C: 13.2		E: constipation (1), dry mouth (1) C: constipation (2), low appetite (3)	E: dizziness (2) C: dizziness (3)	C: dry mouth (1)
Wang et al. (2016)	E: 51 C: 51	Total: 21	Total: 20.59%		Total*: nausea (12)	Total*: dizziness (9)	
Feng et al. (2017)	E: 40 C: 40	E: 0 C: 0	E: 0 C: 0				
Zhang et al. (2020)	E: 41 C: 45	E: 1 C: 2	E: 2.4 C: 4.4	E: palpitations (1)	C: nausea (1)		C: drowsiness (1)
Zhu et al. (2020)	E: 52 C: 52	E: 8 C: 5	E: 15.38 C: 9.62			E: dizziness (3), headache (2) c. dizziness (2), headaches (1)	E: fatigue (3), C: fatigue (2)
Shi et al. (2023)	E: 36 C: 36	E: 13 C: 10	E: 36.11 C: 27.78		E: loss of appetite(2), Nausea(4), diarrhea(1) C: loss of appetite(3), nausea(3)		E: fatigue(2), dizziness(3), dry mouth(1) C: fatigue(1), dizziness(2), dry mouth(1)

*The group in which the participant was involved was not notified

A total of 45 adverse events were reported in the control group. Dizziness was most frequently reported (13 cases), followed by drowsiness (five cases), nausea (six cases), loss of appetite (three cases), fatigue (three cases), dry mouth (two case), headache (one case), and constipation (one case). Most of these were mild or did not require special treatment. However, the trial was discontinued in two cases because of severe dizziness and vomiting [29]. In addition, nausea lasted more than two months, and drowsiness lasted more than one month [35]. A total of 38 adverse events were reported in the treatment group. Dizziness was most frequently reported (15 cases), followed by nausea (six cases), fatigue (five cases), diarrhea (four cases), loss of appetite (two cases), dry mouth (two case), headache (two cases), constipation (one case), and palpitations (one case).

According to a meta-analysis, there was no overall difference in the frequency of adverse effects between SJC and AED or AD. In addition, there was no significant difference in the occurrence of adverse effects between taking the existing AED and adding SJC. However, when AD was added while taking an existing AED, the risk of adverse effects was higher than when SJC was added (OR 2.90, 95% CI: 1.30, 6.48).

Taken together, there was no significant difference in adverse events between the SJC and conventional treatments (OR 1.26, 95% CI: 0.70, 2.26).

### Publication bias

The funnel plot is a tool to investigate publication bias; however, it has limitations when evaluating the effectiveness of small studies of ten or fewer [39]. Since this study included nine studies and all studies were of similar size, publication bias could not be evaluated using funnel plots (fewer than ten studies, Additional File 4).

### Certainty of evidence for main findings

As a result of the GRADE evaluation, the overall certainty of the evidence was critical. All were considered “very low,” and although the effect sizes were large, serious limitations in study design and execution reduced the certainty of the evidence. Additionally, because the number of included papers was less than 10, a publication bias test could not be performed, so all studies were downgraded by one level (Table 2).

**Table 2:** Assessment of certainty of evidence

Outcomes	No. of participants: Control/ Experimental (Studies)	Study design	Risk of bias (high risk of bias: ↓)	Inconsistency (I2 > 50%: ↓)	Indirectness (not clinically assessed: ↓)	Imprecision (CI effect size range > 2: ↓)	presence of publication bias (Yes:	Effect size (> 2.5: ↑)	Dose–response present (↑)	plausible residual confounding present (↑)	Certainty (high, moderate, low, very low)
Depression											
SJC versus conventional treatment	120/120 (2)	RCT	High 2/2 ↓	98% ↓	Not serious	[-0.21, 5.12] ↓	Not detected*	2.45 ↑	No	No	⊕⊖⊖⊖ Very low
SJC plus AED versus conventional treatment	217/219 (5)	RCT	High 5/5 ↓	91%↓	Not serious	[0.28, 1.63]	Not detected*	0.96	No	No	⊕⊖⊖⊖ Very low
SJC plus AED, AD versus conventional treatment	87/87(2)	RCT	High 2/2 ↓	Not applicable**	Not serious	[0.17, 1.57]	Not detected*	0.87	No	No	⊕⊖⊖⊖ Very low
Frequency of seizure											
SJC versus conventional treatment	68/68(1)	RCT	High 1/1 ↓	Not applicable**	Not serious	[0.28, 0.50]	Not detected*	0.39	No	No	⊕⊖⊖⊖ Very low
SJC plus AED versus conventional treatment	149/151(4)	RCT	High 4/4 ↓	87% ↓	Not serious	[-0.28, 0.69]	Not detected*	0.20	No	No	⊕⊖⊖⊖ Very low
Quality of life											
SJC versus conventional treatment	52/52(1)	RCT	High 1/1 ↓	Not applicable **	Not serious	[10.55, 12.95] ↓	Not detected*	11.75 ↑	No	No	⊕⊖⊖⊖ Very low
SJC plus AED versus conventional treatment	77/72(2)	RCT	High 2/2 ↓	95% ↓	Not serious	[-24.10, 8.16] ↓	Not detected*	-7.97 ↑	No	No	⊕⊖⊖⊖ Very low
SJC plus AED, AD versus conventional treatment	87/87(2)	RCT	High 2/2 ↓	Not applicable**	Not serious	[12.34, 21.18] ↓	Not detected*	16.76 ↑	No	No	⊕⊖⊖⊖ Very low
Adverse events											
SJC versus control	338/339(8)	RCT	High 8/8 ↓	19%	Not serious	[0.70, 2.26]	Not detected*	1.26	No	No	⊕⊖⊖⊖ Very low

*Due to fewer than 10 articles were included, no publication bias test was performed. Thus, we downgraded it by one level

** Due to fewer than 2 articles were included, inconsistency cannot be tested. Thus, we downgraded it by one level

## Discussion

### Main findings

This study was a systematic review and meta-analysis investigating the antidepressant effects of the SJC in epilepsy patients. This review was based on nine clinical trials involving 815 patients. Compared to AD, SJC is beneficial in decreasing depression and reducing seizure frequency. In addition, SJC yielded additional advantageous results over AED in terms of antidepressant effects and QoL. Furthermore, there was no significant difference in antidepressant effect, seizure frequency, or QoL indices in either case when SJC or AD was added while continuing AED. However, the risk of adverse effects was higher when AD was added while taking existing AED than when SJC was added. Moreover, while AED and AD are continued, additional SJC administration is more advantageous in terms of antidepressant effects and QoL. There were no reports on seizure frequency.

### Suggestions and values for future clinical practice

These results provide evidence that SJC can be administered to patients who cannot take AED. Even with the development of new AED, 20–30% of epilepsy patients do not respond to treatment [40]. If a patient fails even after two or more AED medication regimens, the remaining viable alternative is surgery, which is highly risky. As there is currently no ultimate treatment for drug-resistant epilepsy, it is expected that SJC will be of interest.

In general, treatment is focused on reducing seizures in patients with epilepsy. However, it has been suggested that depression may have a greater effect on QoL than seizure frequency in treatment-resistant epilepsy [41]. In other words, the treatment of depression should be given more priority. Similarly, depression and seizure frequency simultaneously improved when SJC was used as an adjunct with AED. Although no study has compared seizure frequency when AED versus SJC are taken, this result suggests that prescribing SJC to epilepsy patients can be beneficial for both seizure frequency and depression.

Currently, SSRIs are the most widely prescribed drugs for both epilepsy and depression [42]. It has been reported that SSRIs can reduce symptoms in epilepsy patients with depression. However, patients who received SSRIs were more likely to withdraw owing to the side effects of SSRIs than those without SSRIs [14]. As such, since pooled results showed that SJC had fewer side effects than conventional antidepressants, SJC administration may be considered when there are uncontrolled side effects during AD treatment.

Furthermore, SJC tended to have larger effect sizes in studies with shorter administration periods. In this study, the antidepressant effect of SJC was greater than that of escitalopram at eight weeks when the AED dose was maintained. Although the optimal duration of depression treatment has not been clearly identified, it usually takes two to three weeks for the antidepressant effect to appear [43] and six to nine months for the entire course of treatment [44]. In particular, the control group used escitalopram, which is known to have the quickest antidepressant effect among SSRIs. Only 49–64% of patients treated with escitalopram 10–20 mg/day reported an antidepressant response at the end of 8 weeks [45]. Thus, based on the study finding that SJC had a greater effect than escitalopram in a short period, we confirmed the potential of SJC to rapidly exert greater antidepressant effects than traditional treatment.

A previous meta-analysis evaluated the effect of escitalopram alone over a combination treatment of escitalopram and SJC in patients with major depressive disorder who did not have epilepsy. The combination treatment group showed a better change in HAMD scores before and after treatment, which was also statistically significant (MD = 3.03, 95% CI[-4.59, -1.47], *P* = 0.0001) [17]. In this study, when administered with valproate, the additional administration of escitalopram and SJC also showed superior antidepressant efficacy to additional administration of escitalopram. The change in HAMD scores before and after treatment which was − 1.22 [-1.64, -0.80]) was slightly smaller than that in the study on depression [34]. Despite the various clinical conditions in which depression as an accompanying symptom of epilepsy may differ from major depressive disorders, it can be suggested that valproate can influence SJC. Indeed, some AED (e.g., clobazam and eslicarbazepine) have strong inducing effects on antidepressants, while others (e.g., lacosamide and phenobarbital) act as inhibitors or inducers of antidepressants [46]. Further studies on the drug interactions between SJC and AED are required.

Additionally, according to the results of two studies that evaluated the improvement in anxiety as well as depression by administering SJC to epilepsy patients, SJC can reduce anxiety in patients with epilepsy, which is another challenge in the treatment of epilepsy. Approximately 85% of patients with depression experience severe anxiety symptoms. These patients do not respond well to treatment if they have comorbidities of anxiety and depression [47]. Zhang et al. reported that SJC contributes to reducing agitation and irritability in patients with depression and epilepsy [35]. Therefore, We confirmed the possibility of SJC to complement the limitations of epilepsy treatment in diverse dimensions, especially psychiatric symptoms.

### Suggestions and values for future research

Given that SJC exerts a broad spectrum of beneficial effects on epilepsy, such as antidepressant activity, seizure frequency reduction, and QoL improvement, we can assume the underlying mechanisms of SJC. We expected SJC to influence both depression and seizures.

Modern pharmacological studies on SJC have suggested its’ applicability to patients with epilepsy and depression. Epilepsy and depression share mechanisms associated with hyperactivity of the hypothalamic-pituitary-adrenal axis and impairment of neurotransmitter systems (mainly neurotransmitters serotonin and norepinephrine) [8]. Indeed, it has been reported that SJC and its’ various chemical components modulate the HPA axis, glutamate transport, monoamine transport (norepinephrine, dopamine), BDNF, and other mechanisms [48]. SJC shows antidepressant activity by improving neuronal excitability and increasing the secretion of dopamine and 5-HT [18]. It has also been reported that when a water-based extract of *Acanthopanax senticosus* is administered to mice, the antidepressant mechanism works through the central monoaminergic neurotransmitter system, including 5-hydroxytrilamine (5-HT), norepinephrine (NE), and dopamine (DA) [49]. Chiisanoside, a triterpenoid saponin extracted from *Acanthopanax* senticosus, exhibits antidepressant effects in male ICR mice by modulating monoamine transmission, anti-inflammatory responses, BDNF mechanisms, and glutamate transmission [50]. In addition, it has been reported that hypericin, an active compound of *Hypericum perforatum*, showed antidepressant-like activities through the HPA axis and glutamate transmission in normal and stressed rats [51]. Therefore, SJC and its’ compounds appear to be involved in the common mechanism of depression and epilepsy. However, careful consideration is required because they are involved in a common mechanism. *Hypericum perforatum* can alter the pharmacokinetics of AED by interfering with cytochrome P450 activity [52]. Some of the active compounds of *Acanthopanax senticosus* exert neuroprotection, whereas others may cause potential neurotoxicity [53].

Moreover, it has recently been reported that depression in epilepsy differs from non-epileptic depression on neuroimaging. There is a mechanistic difference, such as a decrease in serotonin availability versus a decrease in serotonin receptors [54]. Therefore, there is a need to develop specific mechanisms and treatments for depression in epilepsy patients. For these patients, if a pharmacological consideration of SJC is made, it will be a potential drug applicable to both epilepsy and depression.

### Strengths and limitations of our research

This review provides crucial evidence that SJC is effective for depression in epilepsy and ameliorates the limitations of antidepressants in epilepsy treatment, such as seizure frequency, QoL, and adverse events. SJC can be considered for depression and mitigate side effects in patients with epilepsy who are using AED or AD. In particular, SJC can be used in depression and for uncontrolled symptoms in patients with refractory epilepsy.

This study has certain limitations. There are limitations in that the results of this study may have been influenced by various factors such as heterogeneity of the evaluation tools, severity of depression, type and dose of antidepressants, and duration of administration. First, although we standardized the results to SMD, heterogeneity remained across the evaluation scales for measuring depression. In terms of the pathological stages of depression, the intervals and stages distinguished by each measure of the PHQ-9, HAMD, CES-D and C-NDDIE were not identical. The PHQ-9 is a self-assessment questionnaire composed of nine indicators of the DSM-IV. The scores are evaluated as follows: no depression (0–4), mild depression [5–9], moderate depression [10–14], and severe depression [15–27, 55]. The HAMD-17 is rated by a clinician and classified as no depression (0–7), mild depression [8–16], moderate depression [17–23], or severe depression (≥ 24) [56]. The CES-D are rated as normal (< 16), mild depression [16–20], moderate depression [21–24], and severe depression (> 25) [57]. The Chinese version of the NDDIE ranges from 6 to 24, with a score of 14 or higher indicating depression [58]. Thus, simply using SMD may not overcome the heterogeneity across studies. Moreover, the degree of baseline depression of the participants in each study varied greatly. In fact, in the inclusion criteria of some studies, the severity of depression was limited to mild and moderate depression, corresponding to 5 to 14 points on the PHQ-9 score [29, 30, 33]. On the other hand, in another study, only patients with moderate or severe depression with a HAMD17 score of 18 or higher were included [36]. As such, before treatment, various ranges of depression were observed in the patient groups. The ranges varied from very mild to severe depression, which can confound the evaluation of efficacy. Moreover, a single dose varied from 20 mg to 1.44 g, and the number of doses per day was not determined to be either once or twice. Since it was reported that twice the dose of SJC increased the antidepressant effect and safely accelerated the reduction of HAMD indicators [19], it is necessary to determine the optimal dosing method through further pharmacokinetic studies. There has been no clear research on the pharmacokinetics and pharmacodynamics tools of SJC, making it difficult to consider the effect size resulting from differences in research duration. Therefore, to justify the different duration of the studies that may influence the result, research on the pharmacokinetics and pharmacodynamics of SJC is necessary. Another limitation of our study is the high degree of heterogeneity observed in many of the meta-analysis results. We attempted to address this issue by conducting a subgroup analysis for the intervention. However, the lack of improvement in heterogeneity even after subgroup analysis indicates the complexity of the factors influencing our study outcomes. We acknowledge that these limitations could impact the interpretation and generalizability of our findings. In addition, the number of included studies was small, methodological quality was low, and heterogeneity was high. As these are all Chinese papers, there may be a publication bias. All the included studies rarely performed blinding. Also, according to the result of GRADE evaluation, the overall certainty of the evidence was critical. To ensure the certainty of evidence in the future, well-designed clinical studies are needed, and a larger number of studies must be secured. Therefore, systematic reviews on this topic should continue to be updated as new studies are published.

## Conclusion

Our study analyzed total eight RCTs that assessed the antidepressant effect of SJC in epilepsy patients. The overall risk of bias was determined to be high in the included studies. In summary, our study suggested that SJC had significant effect than AD in that SJC improved depression and reduced of seizure frequency. Also, comparing to AED, SJC significantly relieved depression and improved quality of life. The additional administration of SJC for AED prescribers could alleviate depression significantly. In terms of limitations, high quality RCTs are needed to encourage our study.

### Electronic supplementary material

Below is the link to the electronic supplementary material.

Supplementary Material 1: PRISMA 2020 Checklist

Supplementary Material 2: Search strategy

Supplementary Material 3: Main characteristics of the included studies

Supplementary Material 4: Funnel plot

## Data Availability

All data analysed during this study are included in this published article.

## Abbreviations

AD: Antidepressants
AED: antiepileptic drugs
CES-D: Center for Epidemiological Studies-Depression Scale
CIs: confidence interval
CINAHL: Cumulative Index to Nursing and Allied Health Literature
C-NDDIE: Chinese-Neurological Disorders Depression Inventory for Epilepsy
DA: dopamine
MDs: mean difference
FST: forced swimming test
NE: norepinephrine
OR: odds ratio
PS: partial seizures
PSE: psychiatric symptoms in epilepsy
PRISMA-P: preferred reporting item for systematic review and meta-analysis
QoL: quality of life
RCTs: randomized clinical trials
REM: rapid eye movement
RRs: relative risks
SDS: Self-Rating Depression Scale
SMDs: standard mean differences
SSRI: selective serotonin reuptake inhibitor
SWS: slow-wave sleep
SJC: Shugan Jieyu capsule
TST: tail suspension test

## References

[1] Michaelis R, Tang V, Nevitt SJ, Wagner JL, Modi AC, LaFrance WC Jr et al. Psychological treatments for people with epilepsy. Cochrane Database of Systematic Reviews. 2020(8). 10.1002/14651858.CD012081.pub3 PMC8409429 35653266

[2] Sridharan R. Epidemiology of epilepsy. Current science. 2002:664 – 70.

[3] Beghi E (2020). The epidemiology of epilepsy. Neuroepidemiology.

[4] Kwan P, Brodie MJ (2000). Early identification of refractory epilepsy. N Engl J Med.

[5] French JA (2007). Refractory epilepsy: clinical overview. Epilepsia.

[6] Hesdorffer DC, Hauser WA, Olafsson E, Ludvigsson P, Kjartansson O (2006). Depression and suicide attempt as risk factors for incident unprovoked seizures. Ann Neurol.

[7] Fiest KM, Dykeman J, Patten SB, Wiebe S, Kaplan GG, Maxwell CJ (2013). Depression in Epilepsy: a systematic review and meta-analysis. Neurology.

[8] Kanner AM. Depression and epilepsy: a bidirectional relation? Epilepsia. 2011;52:21 – 7. 10.1111/j.1528-1167.2010.02907.x 21214536

[9] Danzer SC (2012). Depression, stress, epilepsy and adult neurogenesis. Exp Neurol.

[10] Gandy M, Sharpe L, Perry KN (2012). Psychosocial predictors of depression and anxiety in patients with epilepsy: a systematic review. J Affect Disord.

[11] Jackson M, Turkington D (2005). Depression and anxiety in epilepsy. J Neurol Neurosurg Psychiatry.

[12] Mehndiratta P, Sajatovic M (2013). Treatments for patients with comorbid epilepsy and depression: a systematic literature review. Epilepsy Behav.

[13] Pisani F, Spina E, Oteri G (1999). Antidepressant drugs and seizure susceptibility: from in vitro data to clinical practice. Epilepsia.

[14] Maguire MJ, Marson AG, Nevitt SJ. Antidepressants for people with epilepsy and depression. Cochrane Database of Systematic Reviews. 2021(4). 10.1002/14651858.CD010682.pub3 PMC8094735 33860531

[15] Barry JJ, Huynh N, Lembke A (2000). Depression in individuals with epilepsy. Curr Treat Options Neurol.

[16] Yao G, Li J, Wang J, Liu S, Li X, Cao X (2020). Improved resting-state functional dynamics in post-stroke depressive patients after shugan jieyu capsule treatment. Front NeuroSci.

[17] Shuai M, Min J, Xiaoyan X (2021). Meta-analysis of Shugan Jieyu Capsule combined with Escitalopram in the treatment of depression. J Shandong First Med Unversity Shandong Acad Med Sci.

[18] Fan M, Guo D, Tian Y, Liu Y, Zhao J. Efficacy and safety of Shugan Jieyu capsule in the treatment of essential hypertension with insomnia, anxiety or depression: a protocol for systematic review and meta-analysis. Medicine. 2021;100(8). 10.1097/MD.0000000000024856 PMC7909162 33663107

[19] LI Q, Yao J, WU W, YU X, Wang W, HU Y et al. Efficacy and safety of double doses of SHUGANJIEYU capsules in treating moderate depressive disor-der: a multicenter, random, double-blind, and parallel-controlled trial. Chin J Nerv Mental Dis. 2016:580–5.

[20] Ng QX, Venkatanarayanan N, Ho CYX (2017). Clinical use of Hypericum perforatum (St John’s wort) in depression: a meta-analysis. J Affect Disord.

[21] Linde K, Berner MM, Kriston L. St John’s wort for major depression. Cochrane Database of Systematic Reviews. 2008(4). 10.1002/14651858.CD000448.pub3 PMC7032678 18843608

[22] Ernst E, Rand J, Barnes J, Stevinson C (1998). Adverse effects profile of the herbal antidepressant St. John’s wort (Hypericum perforatum L). Eur J Clin Pharmacol.

[23] Rezaei A, Rezaei-Dorostkar K, Ahmadizadeh C, Jafari B. A comparative study of sedative and anxiolytic effects of the Hypericum perforatumin and diazepam on rats. Zahedan J Res Med Sci. 2012;13(8).

[24] Hosseinzadeh H, Karimi G-R, Rakhshanizadeh M (2005). Anticonvulsant effect of Hypericum perforatum: role of nitric oxide. J Ethnopharmacol.

[25] Ivetic V, Popovic M, Mimica-Dukic N, Barak O, Pilija V (2002). St. John’s wort (Hypericum perforatum L.) and kindling epilepsy in rabbit. Phytomedicine.

[26] Sterne JA, Savović J, Page MJ, Elbers RG, Blencowe NS, Boutron I et al. RoB 2: a revised tool for assessing risk of bias in randomised trials. BMJ. 2019;366. 10.1136/bmj.l4898 31462531

[27] Patsopoulos NA, Evangelou E, Ioannidis JP (2008). Sensitivity of between-study heterogeneity in meta-analysis: proposed metrics and empirical evaluation. Int J Epidemiol.

[28] Egger M, Smith GD, Schneider M, Minder C (1997). Bias in meta-analysis detected by a simple, graphical test. BMJ.

[29] Liang Y, Yulan H, Bin H, Baoming H, Suping L, poplar (2015). A comparative study on shugan-jieyu capsule and sertraline in the treatment of epilepsy patients combined with mild to moderate depression practical. Hosp Clin J.

[30] Yulan H, Liang Y, Yi H (2015). Effects of Shugan-Jieyu Capsule on mild to Moderate Depression in adult patients with epilepsy. J Chengdu Med Coll.

[31] Xiaoling Z (2020). Effects of Shugan Jieyu capsules on patients with epilepsy and depressive disorder. China Minkang Medicine.

[32] Jiaping X, Zhiru Z, Xiongfei Z, Ruijuan Z (2015). Therapeutic effect of Shugan Jieyu capsule in the treatment of epilepsy complicated with depression. Int J Psychiatry.

[33] Xingmei F, Chunxia L (2017). Analysis of the effect of comprehensive nursing intervention on Shuganjieyu capsule in the treatment of patients with post-epilepsy depression and its optimization and improvement. Disease Surveillance and Control.

[34] Hongshan P, Cuiying M, Jiayi H (2016). Shuganjieyu Capsule in the treatment of depressive symptoms in patients with Epilepsy: evaluation of clinical efficacy and safety. Chin folk Med.

[35] Chenqi Z, Meiling H, Hongbin S (2020). The efficacy,safety and quality of life of tandospirone and shuganjieyu capsule in the treatment of comorbid anxiety and depression of epilepsy. Practical Hosp Clin J.

[36] Xiaorong W, Xiaolan L, Ya C, Qionggui Z (2016). Analysis of the effect of comprehensive nursing intervention on Shuganjieyu capsule in the treatment of patients with post-epilepsy depression and its optimization and improvement. World Clin Med.

[37] Huan S, Bo L (2023). The efficacy of tandospirone citrate combined with Shugan Jieyu capsule in the treatment of anxiety and depression comorbid with epilepsy. Ration Clin use Drugs.

[38] Hong-bin ZC-qHM-lS (2020). The efficacy, safety and quality of life of tandospirone and shuganjieyu capsule in the treatment of comorbid anxiety and depression of epilepsy. Practical Hosp Clin J.

[39] Sterne JA, Sutton AJ, Ioannidis JP, Terrin N, Jones DR, Lau J et al. Recommendations for examining and interpreting funnel plot asymmetry in meta-analyses of randomised controlled trials. BMJ. 2011;343. 10.1136/bmj.d4002 21784880

[40] Dalic L, Cook MJ (2016). Managing drug-resistant epilepsy: challenges and solutions. Neuropsychiatr Dis Treat.

[41] Boylan L, Flint L, Labovitz D, Jackson S, Starner K, Devinsky O (2004). Depression but not seizure frequency predicts quality of life in treatment-resistant epilepsy. Neurology.

[42] Kondziella D, Asztely F (2009). Don’t be afraid to treat depression in patients with epilepsy!. Acta Neurol Scand.

[43] Taylor MJ, Freemantle N, Geddes JR, Bhagwagar Z (2006). Early onset of selective serotonin reuptake inhibitor antidepressant action: systematic review and meta-analysis. Arch Gen Psychiatry.

[44] Hathaway EE, Walkup JT, Strawn JR (2018). Antidepressant treatment duration in pediatric depressive and anxiety disorders: how long is long enough?. Curr Probl Pediatr Adolesc Health Care.

[45] Lam RW, Annemans L (2007). Efficacy, effectiveness and efficiency of escitalopram in the treatment of major depressive and anxiety disorders. Expert Rev PharmacoEcon Outcomes Res.

[46] Italiano D, Spina E, de Leon J (2014). Pharmacokinetic and pharmacodynamic interactions between antiepileptics and antidepressants. Expert Opin Drug Metab Toxicol.

[47] Gorman JM (1996). Comorbid depression and anxiety spectrum disorders. Depress Anxiety.

[48] Zhang M, Bai X. Shugan Jieyu Capsule in Post-stroke Depression Treatment: from molecules to systems. Front Pharmacol. 2022:61. 10.3389/fphar.2022.821270 PMC8818889 35140618

[49] Jin L, Wu F, Li X, Li H, Du C, Jiang Q (2013). Anti-depressant effects of Aqueous Extract from Acanthopanax senticosus in mice. Phytother Res.

[50] Bian X, Liu X, Liu J, Zhao Y, Li H, Cai E (2018). Study on antidepressant activity of chiisanoside in mice. Int Immunopharmacol.

[51] Butterweck V, Winterhoff H, Herkenham M (2001). St John’s wort, hypericin, and imipramine: a comparative analysis of mRNA levels in brain areas involved in HPA axis control following short-term and long-term administration in normal and stressed rats. Mol Psychiatry.

[52] Spinella M (2001). Herbal medicines and epilepsy: the potential for benefit and adverse effects. Epilepsy Behav.

[53] Zhang S-n (2014). Neuroprotection or neurotoxicity? New insights into the effects of Acanthopanax senticosus harms on nervous system through cerebral metabolomics analysis. J Ethnopharmacol.

[54] Elkommos S, Mula M (2021). A systematic review of neuroimaging studies of depression in adults with epilepsy. Epilepsy Behav.

[55] Manea L, Gilbody S, McMillan D (2012). Optimal cut-off score for diagnosing depression with the Patient Health Questionnaire (PHQ-9): a meta-analysis. CMAJ.

[56] Ma S, Yang J, Yang BX, Kang L, Wang P, Zhang N et al. The Patient Health Questionnaire-9 versus the Hamilton Rating Scale for Depression in assessing major depressive disorder. Front Psychiatry. 2021:1974. 10.3389/fpsyt.2021.747139 PMC8599822 34803766

[57] Kohout FJ, Berkman LF, Evans DA, Cornoni-Huntley J (1993). Two shorter forms of the CES-D depression symptoms index. J Aging Health.

[58] Xia N-g, Ding S-q, Lin J-h, Dong F-r, Du Y-r, Wang X-s (2020). Comparison of the performance of two depression rating scales in patients with epilepsy in southern China. Epilepsy Behav.

