# Supplementary material for: Effectiveness of Shugan Jieyu capsules for psychiatric symptoms of epilepsy: a systematic review and meta-analysis
Source: BMC Complement Med Ther. 2024 Jan 29;24:63. doi: 10.1186/s12906-024-04361-0 (PMC10825991; doi:10.1186/s12906-024-04361-0)
Supplement: Supplementary file 2 — Supplementary Material 2: Search strategy [file 12906_2024_4361_MOESM2_ESM.docx]

**Additional file 2. Search strategy**

**MEDLINE (PubMed)**

|  | Keywords | Search strategy |
| --- | --- | --- |
| #1 | Epilepsy keywords | ("epilepsy"[Mesh] OR epilep*[Title/Abstract] OR "seizures"[Mesh] OR “convulsion”[Title/Abstract] OR seizure*[Title/Abstract]”) |
| #2 | Shugan Jieyu capsule keywords^†^ | “Shugan jieyu” [Title/Abstract] OR “Shugan jieyu capsule”[Title/Abstract] OR “Shuganjieyu”[Title/Abstract] OR “Shugan-jieyu”[Title/Abstract] OR {(“Acanthopanax senticosus”[tw] OR “Eleutherococcus senticosus”[tw] OR “Siberian Ginseng”[tw]) OR (“Hypericum perforatum”[tw] OR “John's wort”[tw])} |
| #3 |  | #1 AND #2 |

^†^ The original search strategy includes the similar search terms in Chinese language as repeated from those in English language but it was not unable to reported in the additional file due to the regulations of the journal. The original search strategy is available upon request.

**EMBASE**

|  | Keywords | Search strategy |
| --- | --- | --- |
| #1 | Epilepsy keywords | 'seizure, epilepsy and convulsion'/exp/mj |
| #2 |  | ‘epilep*’ |
| #3 |  | ‘seizures’ |
| #4 |  | ‘convulsion’ |
| #5 |  | ‘seizure*’ |
| #6 | Shugan Jieyu capsule keywords^†^ | Shugan AND jieyu |
| #7 |  | Shugan AND jieyu AND capsule |
| #8 |  | ‘Shuganjieyu’ |
| #9 |  | ‘Shugan-jieyu’ |
| #10 |  | ('acanthopanax senticosus' OR 'eleutherococcus senticosus' OR 'siberian ginseng') AND ('hypericum perforatum' OR 'johns wort') |
| #11 |  | (#1 OR #2 OR #3 OR #4 OR #5) AND (#6 OR #7 OR #8 OR #9 OR #10 OR #11) |

^†^ The original search strategy includes the similar search terms in Chinese language as repeated from those in English language but it was not unable to reported in the additional file due to the regulations of the journal. The original search strategy is available upon request.

**The Cochrane Central Register of Controlled Trials (CENTRAL)**

|  | Keywords | Search strategy |
| --- | --- | --- |
| #1 | Epilepsy keywords | (Epilepsy) OR (epilep*) OR (seizures) OR (convulsion) OR (seizure*) in Title Abstract Keyword AND (Shugan jieyu) OR (Shugan jieyu capsule) OR (Shuganjieyu) OR (Shugan-jieyu) OR ((acanthopanax senticosus) OR (eleutherococcus senticosus) OR (siberian ginseng)) |
| #2 | Shugan Jieyu capsule keywords | ((hypericum perforatum) OR (john’s wort)) |
| #3 |  | #1 AND #2 |

**Cumulative Index to Nursing and Allied Health Literature (CINAHL)**

|  | Keywords | Search strategy |
| --- | --- | --- |
| #1 | Shugan Jieyu capsule keywords | Shugan jieyu |
| #2 |  | Shugan jieyu capsule |
| #3 |  | ‘Shuganjieyu’ |
| #4 |  | ‘Shugan-jieyu’ |
| #5 |  | ('acanthopanax senticosus' OR 'eleutherococcus senticosus' OR 'siberian ginseng') AND ('hypericum perforatum' OR 'john’s wort') |
| #6 |  | #1 OR #2 OR #3 OR #4 OR #5 |

**Korean Medical Database (KMBASE)**

|  | Keywords | Search strategy |
| --- | --- | --- |
| #1 | Shugan Jieyu capsule keywords^†^ | ([ALL=Shugan jieyu]) |

^†^ The original search strategy includes the similar search terms in Korean language as repeated from those in English language but it was not unable to reported in the additional file due to the regulations of the journal. The original search strategy is available upon request.

**OASIS, Korean Traditional Knowledge Portal**

|  | Keywords | Search strategy |
| --- | --- | --- |
| #1 | Shugan Jieyu capsule keywords^†^ | ‘shugan-jieyu’ |

^†^ The original search strategy includes the similar search terms in Korean language as repeated from those in English language but it was not unable to reported in the additional file due to the regulations of the journal. The original search strategy is available upon request.

**CiNii**

|  | Keywords | Search strategy |
| --- | --- | --- |
| #1 | Shugan Jieyu capsule keywords^†^ | ('Shugan Jieyu' OR 'Shugan Jieyu Capsule’) |
| #2 |  | ('Hypericum perforatum' AND ('Acanthopanax senticosus' OR 'Eleutherococcus senticosus')) |
| #3 |  | #1 OR #2 |

^†^ The original search strategy includes the similar search terms in Japanese language as repeated from those in English language but it was not unable to reported in the additional file due to the regulations of the journal. The original search strategy is available upon request.

**China National Knowledge Infrastructure (CNKI).**

|  | Keywords | Search strategy |
| --- | --- | --- |
| #1 | Epilepsy keywords | AB=('Shugan jieyu'+'Shugan jieyu capsule'')+('Hypericum perforatum')*('Acanthopanax senticosus' + 'Eleutherococcus senticosus') |
| #2 | Shugan Jieyu capsule keywords^†^ | AB=('epilepsy'+'epilep*'+ 'seizure ‘+’Epileptic Syndrome'+'Spasm'+'convulsion') |
| #3 |  | #1 AND #2 |

^†^ The original search strategy includes the similar search terms in Chinese language as repeated from those in English language but it was not unable to reported in the additional file due to the regulations of the journal. The original search strategy is available upon request.
