# Supplementary material for: Effectiveness of Shugan Jieyu capsules for psychiatric symptoms of epilepsy: a systematic review and meta-analysis
Source: BMC Complement Med Ther. 2024 Jan 29;24:63. doi: 10.1186/s12906-024-04361-0 (PMC10825991; doi:10.1186/s12906-024-04361-0)
Supplement: Supplementary file 3 — Supplementary Material 3: Main characteristics of the included studies [file 12906_2024_4361_MOESM3_ESM.docx]

**Additional File 3.** Main characteristics of the included studies

| **First author (publication year)** | **Sample size** | **Age range or mean age** | **Duration of disease (epilepsy); Type of seizure** | **Diagnosis** | **Baseline Depression scale** | **Baseline Epilepsy scale** | **Baseline QoL scale** | **Experimental group** | **Control group** | **Treatment period** | **Outcome measurements** | **Adverse Events (n)** |
| --- | --- | --- | --- | --- | --- | --- | --- | --- | --- | --- | --- | --- |
| Huang et al (2015) | E: 32  C: 31 | E: 18-65 yr, mean 31.23±11.71 yr  C: 19-71 yr, mean 30.72±12.45 yr | E: 1-12 yr, mean 6.57±3.74 yr; PS(20), GS(12)  C: 1-13 yr, mean 6.04±4.04 yr; PS(18), GS(13) | 1. ILAE 1981  2. ILAEc 1989  3. PHQ-9 : 5-14 | E: 8.43±2.61  C: 8.71±2.81 | E: 1.71±0.57  C: 1.86±0.75 | E: 50.00±10.84  C: 50.20±10.35 | Shugan Jieyu capsule 1.44g (2 times/day) + Antiepileptic drugs | Antiepileptic drugs (31) | 12 wk | 1. PHQ-9 2. Frequency of seizures 3. QOLIE-31  4. Electrocardiogram, blood, urine, liver and kidney function | E: nausea(1), dizziness(1), diarrhea (1)  C: dizziness(1), drowsiness (1) |
| Xu et al (2015) | E: 68  C: 69 | 22-67 yr, mean 41.3 ± 6.2 yr | 2-14 yr, mean 7.0 ± 1.2 yr ; PS(84), GS(52) | 1. ILAE 1981  2. Depression in CCMD-3 | E: 35. 46±3. 12  C: 35. 52±3. 15 | E: 1. 97±0. 34  C: 2. 01±0. 36 | Not mentioned. | Shugan Jieyu capsule 0.72g (2 times/day) | Antidepressants, EEG therapy, music therapy, psychotherapy | 12 wk | 1. HAMD  2. Frequency of seizures 3. QOLIE-31 | E: Diarrhea(2), nausea(1), dizziness(1)  C: Diarrhea (1), nausea(2), dizziness(2) |
| Yu et al (2015) | E: 38  C: 33 | E: 18-72yr, mean 37. 00 ± 21. 01 yr  C: 18-69yr, mean 36.65±20.74 yr | E: 0.7-1.5yr, mean 5.75±3.07 yr; PS(24), GS(14)  C: 1-13 yr, mean 6.13±3.48 yr; PS(22), GS(14) | 1. ILAE 1981  2. ILAEc 1989  3. PHQ-9 : 5-14 | E: 10. 63±3. 16  C: 11. 25±2. 60 | E: 1. 50±0. 53  C: 1. 63±0. 69 | Not mentioned. | Shugan Jieyu capsule 1.44g (2 times/day) + Antiepileptic drugs | Sertraline 100mg (1 times/day) + Antiepileptic drugs | 24 wk | 1. PHQ-9 2. Frequency of seizures  3. Electrocardiogram, blood, urine, liver and kidney function | E: minor adverse reactions such as dizziness, nausea, and diarrhea (5)  C: withdrawn due to severe dizziness and vomiting(2), mild dizziness(3), dry mouth(2), lethargy(3), diarrhea(1), constipation(1)  (p< 0.05). |
| Pan et al (2016) | E: 68  C: 68 | E: 23-63 yr, mean 43.1±5.5 yr,  C: 24-61 yr, mean 42. 7 ± 5. 9 yr | E: 1-10 yr, mean 4.9±1.4 yr; PS(36), GS(32)  C: 1-9 yr, mean 5. 1 ± 1.6 yr ; PS(35), GS(33) | 1. ILAE 1981  2. Depression in ICD-10 | E: 25. 1 ± 3.4  C: 25. 3 ± 3.3 | Not mentioned. | Not mentioned. | Shugan Jieyu capsule 0.72g (2 times/day) + Valproate Sodium 20-30 mg/kg (1 times/day) + Lamotrigine 25mg (1 times/day) | Escitalopram 10mg (1 times/day) + Valproate Sodium 20-30 mg/kg (1 times/day) + Lamotrigine 25mg (1 times/day) | 8 wk | 1. HAMD  2. Self-Rating Depression Scale (SDS)  3. TESS Adverse Reaction Scale | E: Dizziness (2), Constipation (1), Dry mouth (1)  C: Dizziness (3), Constipation (2), Dry mouth (1), Anorexia (3)  (p<0.05) |
| Wang et al (2016) | E: 51  C: 51 | 15 - 68 yr  E: mean 26.6±12.8 yr  C: mean 22.2±16.3 yr | NR | 1. Epilepsy through clinical and laboratory tests [including EEG, CT or MRI]  2. Depression in CCMD-3  3. HAMD17 : > 18 pts | E: 34.2±10.1  C: 33.7±9.9 | Not mentioned. | E: 35.2±5.1  C: 34.7±9.9 | Shugan Jieyu capsule 20mg (1 times/day) + Antiepileptic drugs  + Paroxetine 20mg (1 times/day) | Paroxetine 20mg, 40mg* (1 times/day)  + Antiepileptic drugs | 24 wk | 1. HAMD  2. QOLIE-31 | Gastrointestinal reactions, including mild nausea and bloating (12), dizziness (9). |
| Feng et al (2017) | E: 40  C: 40 | E: 23-57 yr, mean 31.81 ± 6.92 yr  C: 21-56 yr, mean 32.31 ± 6.88 yr | E: 1-10 yr, mean 6.15 ± 3.43 yr; PS(25), GS(15)  C: 1-10 yr, mean 6.13 ± 3.20 ; PS(26), GS(14) | 1. Epilepsy (the criteria are not specified)  2. PHQ-9 : 5-14 | E: 8.78±2.58  C: 8.83±2.61 | E: 1.78±0.68  C: 1.83±0.66 | Not mentioned. | Shugan Jieyu capsule 0.36g (2 times/day) + Routine nursing care  + Antiepileptic drugs | Routine nursing care  + Antiepileptic drugs | 12 wk | 1. PHQ-9 2. Frequency of seizures  3. QOLIE-31 | NR |
| Zhang et al (2020) | E: 41  C: 45 | 18 - 70 yr  E: 34. 37±13. 59 yr  C: 32. 07±11. 28 yr | E: mean 9. 61±9. 21 yr; PS(10), GS(25), PS+GS(5), Unknown(1)  C: mean 7. 54±7. 93 yr; PS(14), GS(29), PS+GS(2) | 1. ILAE 2017  2. Depression and Anxiety in ICD-10  3. C-NDDIE > 12 pts  4. GAD-7 > 6pts | E: 13.35±1. 70  C: 13.22±1. 74 | E: 3.76±3.87  C: 2.58±2.76 | E: 50.81± 10.03  C: 51.50± 13.94 | Shugan Jieyu capsule 0.36g (2 times/day) + Antiepileptic drugs | Tandospiron 10mg + Antiepileptic drugs | 12 wk | 1. C-NDDIE 2. GAD-7  3. Frequency of seizures  4. QOLIE-31 | E: palpitations and discomfort(1)  C: drowsiness for about 1 month(1), 2 months with mild nausea, vomiting and other gastrointestinal reactions(1) |
| Zhu (2020) | E: 52  C: 52 | E: 20-68 yr, mean 48.25±1.49 yr  C: 22-70 yr, mean 48.12±1.56 yr | E: 1-12 yr, mean 6.92±2 yr  / Depressive disorder: 4-10 wk, mean 8.12±1.18 wk  C: 2- 10 yr, mean 6.96± 0.48 yr  /Depressive disorder: 3-10 wk, mean 8.16±1.15 wk | 1. 2004 Chinese epilepsy diagnosis guidelines  2. Depressive Disorder less than 3 months | E: 24.92±7.16  C: 24.85±7.12 | Not mentioned. | E: 36.98±2.75  C: 36.72±2.96 | Shugan Jieyu capsule 0.36g (2 times/day) | oxcarbazepine 0.6g** (2 times/day) | 18 wk | 1. HAMD  2. QOLIE-31 | E: dizziness (3), fatigue (3), headache (2)  C: dizziness (2), fatigue (2), headache (1) |
| Shi  (2023) | E: 36  C: 36 | E:45-73yr, mean 60.2±9.7yr  C: 46-72yr  Mean 59.4±9.3yr | E: 6-34 month, mean 16.8±8.2 month  C: 6-32 month, mean 15.4±8.4 month | 1.Chinese epilepsy diagnosis guideline  2. Epilepsy confirmed through electroencephalography and magnetoencephalography  3. Depression and anxienty confirmed by a psychiatrist | E: 43.43±12.17  C: 42.61±12.45 | Not mentioned. | E: 51.48±10.23  C: 51.95±9.75 | Shugan Jieyu capsule 0.72g (2 times/day) + Antiepileptic drugs  + Tandospirone citrate 10mg (3 times/day) | Antiepileptic drugs  + Tandospirone citrate 10mg (3 times/day) | 2 wk | 1. CES-D  2. GAD-7 3. QOLIE-31 | E:  loss of appetite(2), Nausea(4), diarrhea(1), fatigue(2), dizziness(3), dry mouth(1)  C: loss of appetite(3), nausea(3), fatigue(1), dizziness(2), dry mouth(1) |

PSE = Psychiatric symptoms in epilepsy, E = experimental group, C = control group, NR = not reported, QOLIE-31 = Quality of life in epilepsy—31 inventory, C-NDDIE = Chinese-Neurological Disorders Depression Inventory for Epilepsy, GAD-7 = The Generalized Anxiety Disorder scale-7., PS = partial seizures, GS = generalized seizures, ILAE 1981 = the 1981 International Antiepileptic Federation (ILAE) diagnostic criteria for epilepsy, ILAEc 1989 = the 1981 International Antiepileptic Federation (ILAE) classification criteria for epilepsy, ILAE 2017 = the 2017 International Antiepileptic Federation (ILAE) diagnostic criteria for epilepsy according to clinical symptoms and electroencephalogram (EEG) changes , CCMD-3 = Chinese Classification and Diagnosis of Mental Disorders : Standard 3rd Edition

*Among them, 18 patients adjusted the dose to 40 mg once a day after 2 weeks of treatment due to poor efficacy.

** Initial dose is 0.6 g, but can be increased every week depending on the patient (maximum 2.4 g/day)
