# Supplementary figures and images for: Effectiveness of Shugan Jieyu capsules for psychiatric symptoms of epilepsy: a systematic review and meta-analysis
Source: BMC Complement Med Ther. 2024 Jan 29;24:63. doi: 10.1186/s12906-024-04361-0 (PMC10825991; doi:10.1186/s12906-024-04361-0)

## Additional File 4. Funnel plot

### A) Depression

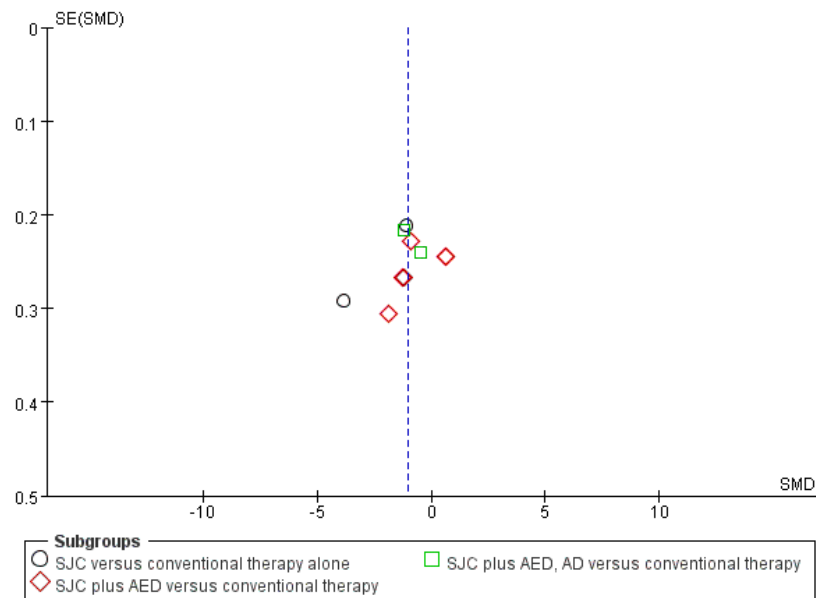

### B) Seizure frequency

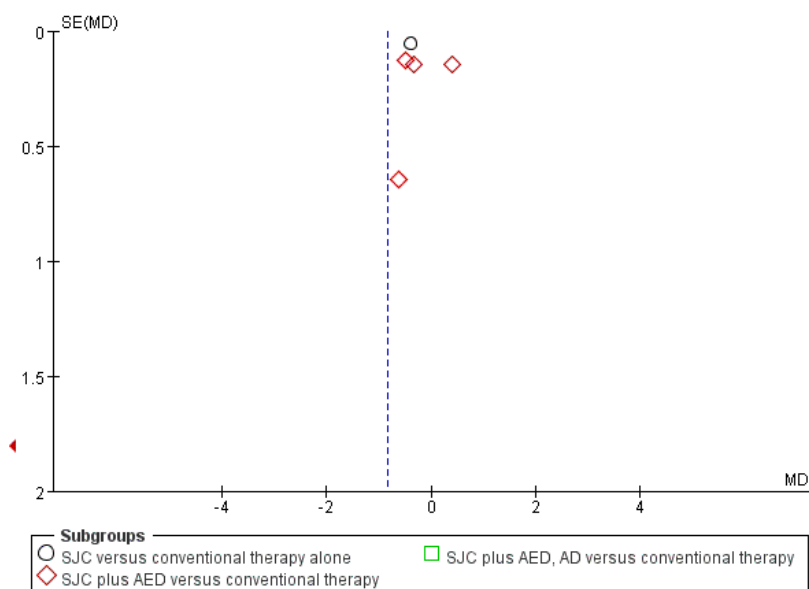

Supplement: Supplementary file 4 — Supplementary Material 4: Funnel plot [file 12906_2024_4361_MOESM4_ESM.pdf]
